# OD29 From Listening To Lift Off: Developing A Three-Year Public Involvement And Engagement Strategy For NICE

**DOI:** 10.1017/S0266462324001600

**Published:** 2025-01-07

**Authors:** Mark Rasburn, Helen Crosbie, Ella Fitzpatrick, Heidi Livingstone, Clare Morgan, Laura Norburn, Sally Taylor, Mandy Tonkinson, Janine Wigmore

## Abstract

**Introduction:**

Involving patients in the health technology assessment (HTA) lifecycle is a core principle at the National Institute for Health and Care Excellence (NICE). To achieve this, NICE has adopted a mixed approach to patient and public involvement and engagement (PPIE) spanning the entire appraisal process. To ensure the PPIE approach enables meaningful involvement, NICE engaged with stakeholders to review its effectiveness and identify areas for improvement.

**Methods:**

In 2023, an independent consultant reviewed NICE’s PPIE approach and engaged with NICE staff and external stakeholders from patient organizations, individual patient contributors, and engagement leads at national health and social care organizations. The engagement included interviews with NICE staff (n=19) and external stakeholders (n=13), and an online survey that received 83 responses from patient organizations and patient contributors. Using this feedback, NICE’s patient and public involvement program conducted four focus groups to develop a framework of improved methods and processes for PPIE with NICE staff, patient organizations, and patient contributors.

**Results:**

The engagement identified many positives in NICE’s approach to PPIE, including:

- lay members sitting on each HTA committee as equal members
- patient organizations providing written evidence to HTA committees
- patient experts providing written and verbal testimony to HTA committees
- support provided by NICE.

The engagement also identified areas where PPIE could have a greater impact, including:

- improved methods for collecting patient evidence and insight
- strengthening the role of lay members
- collating and reusing previously collected patient evidence
- taking a proportionate approach to involving small organizations
- allocating staff resources to focus on impactful PPIE practices.

**Conclusions:**

NICE has developed a draft framework for an improved approach to increase the impact of PPIE in HTA decision-making. In 2024, NICE will publicly consult with NICE staff and external stakeholders to review the framework, agree the strategic aims, and develop metrics for measuring success. Following this consultation, the findings and NICE’s updated approach to PPIE will be presented.

